# Supplementary material for: Elucidating the effect of mechanical stretch stress on the mechanism of ligamentum flavum hypertrophy: Development of a novel in vitro multi-torsional stretch loading device
Source: PLoS One. 2022 Oct 21;17(10):e0275239. doi: 10.1371/journal.pone.0275239 (PMC9586365; doi:10.1371/journal.pone.0275239)
Supplement: Attachment — Submitted filename: Answer to reviewers_v1.2.docx [file pone.0275239.s002.docx]

Answer to reviewers’ comments

Reviewer #1

1. Negative data should be demonstrated in Table 1

Thank you for the comment that needs to be revised. We have added the negative value of MMP-9 and TIMP to the Table 1, accordingly.

2. Write the full name of MSS when it appears at the first time in the Abstract.

Thank you for your comment. We have corrected the abstract accordingly to ‘Stretch strength of the device was optimized by applying 5% and 15% mechanical stretch stress (MSS) loads for 24, 48, and 72 h.’ [Line27,28]

3. The phenotype of cell hypertrophy is an important concept in this research but there are no direct findings to clarify this pathology in the present study.

Thank you for your comment that is worth discussing. While it would be also valuable to demonstrate the phenotypic characteristics at the cellular level, ligamentum hypertrophy occurs in constant and aberrant remodeling of extracellular matrix. The authors demonstrated the change in cytokines, enzymes, and growth factors that is associated with aberrant remodeling of ECM by applying stressful conditions to the LF cells that mimic in vivo mechanical stress.

Reviewer #2:

1. Why is the cell density of 1 X10^4 /mL used? It seems not growth confluence on the membrane of the device.

Thank you for your comment. We apologize for the mistyping in the manuscript. It is supposed to be 1x10^5/mL and we have corrected it accordingly as follows;

‘LF cells were plated on the PDMS chamber at a density of 5.0 × 10^4^ /mL.’ [Line 127]

2. 2. How is the percentage of stretch force determined? What’s the meaning of 5%, or 15%? Does it mean that there is a setting of 100%? What’s the scale?

We greatly appreciate the reviewer for pointing out this ambiguity in explaining the definition of each MSS strength percentages. The 5% and 15% in this study is the strength compared to the possible maximal stretch force which can be generated by our new MSS device. So, it is the relative magnitude of MSS strength. As this is an in vitro study, we still cannot provide information regarding the relative strength compared to the in vivo physiologic mechanical stress. The setting of the magnitude of stretch strength is explained in Line 129-131

3. . Why are two kinds of statistical analysis for the p value? Why is different manner used?

Thank you for your comment which may have caused confusion. Two different statistical methods were used due to difference in normality distributions, which was conducted by Shapiro-Wilk test. In order to cause less confusion we corrected the corresponding sentence to ‘P values were calculated using Student’s t-test or the Mann–Whitney U test, as appropriate according to sample size and distribution normality determined by Shapiro-Wilk test. [Line 140-142]

4. In result section of cytotoxicity assay of LF cells, no 72 h of results shows. I am confused that the time duration of 24, 48 or 72 h. Does it mean that the cells are torsional stress for 24, 48 or even 72 h? Or cells are torsional stress for 5 or 10 min and rest for 24, 48 or 72h? It seems not reasonable to torsional stress cells for such long time duration.

Thank you for your comment. The authors have removed the 72hr stress from the figure, as 48hr stress already demonstrated the significant cytotoxicity. But we fully agree with the reviewer, that this may have created some confusions. Therefore, we added the 72hr stress to the figure. The purpose of this section of the study was to test the cellular viability of the experiment. Applying 5% stress for 24 hr seem not to cause cell death, as demonstrated by cytotoxic assay that does not (or insignificantly) differ from that of control. Therefore, we considered 24hr duration of stretch-torsional stress to be suitable for the subsequent experiment.

5. It’s a suggestion to test cell viability by one more assay experiment such as MTT assay.

Thank you for your comment. It is true that additional assay such as MTT may strengthen the cellular viability test. However, it is technically difficult at the moment as all experiments are already carried out. In the subsequent studies, the authors will consider carrying out the experiment. We have added following sentence in the discussion as part of limitation of this study; ‘Further studies concerning intracellular signaling RNA with additional assay experiment, such as MTT assay, and various cell staining technique to validate cytoskeletal changes and cellular viability might lead us to a more abundant conclusion regarding the bio-mechanism.’ [Line 273-275]

6. The experiments of force stress with different strength loading should be tested.

Thank you for your comment. Carrying out experiments with different loading stress would of course strengthen the data and the authors fully agree with your opinion. However, this is a pilot study to test out the novel device. In the future studies, along with additional viability test, multiple other loading stresses will be applied as suggested. We have added following sentence in the discussion as part of limitation of this study; ‘In order to do so, in vivo and further follow up experimental studies with more various loading stress are needed.’ [Line 277,278]

7. Are the control group pictures of fig 3B and 3C the same? How long is the control duration? 0, 24, 48 or 72 h? In addition, a live and dead cell staining and a cytoskeleton change staining will be more suitable to prove the change of cell morphology.

Thank you for your comment. There was a mistake during the image frame-working. We have corrected it with the appropriate image. We fully agree and appreciate with your opinion of staining the cells. However, at this stage of experiment it is not feasible to do so. We will use the cell staining technique in our follow-up studies We have added following sentence in the discussion as part of limitation of this study; ‘Further studies concerning intracellular signaling RNA with additional assay experiment, such as MTT assay, and various cell staining technique to validate cytoskeletal changes and cellular viability might lead us to a more abundant conclusion regarding the bio-mechanism.’ [Line 273-275]

8. The rationale of inlammation or ECM marker tested in this manuscript should be provided in introduction seciton.

Thank you for your comment. The authors agree with your comment that it is worth mentioning the rationale behind the markers used in this study. We used the following literatures to support the rationale for markers used; [Moon et al., 2012, *Spine*, Hur et al., 2015, *Neurosurgery*, Kim et al., 2016, J Neurosurg Spine]. We have added following sentence to the introduction section; ‘We evaluated the molecular biological responses related to inflammation, angiogenesis, and extracellular matrix (ECM) regulation that were reported in previous studies of ligamentum flavum cells to various stress loads to identify the stress load that best mimics LFH.’ [Line 67,68]

9. the manuscript would be better to be revised by a naive speaker.
   Thank you for your comment. It is true for us – second language speakers – to leave the manuscript for native speakers to revise. We have used ‘Textcheck’ to revise the manuscript. This is a professional English consulting site for editing documents for publication in international journals. The website and reference number are http://www.textcheck.com/text/page/times and 21052202, respectively.
