# Supplementary material for: Elucidating the effect of mechanical stretch stress on the mechanism of ligamentum flavum hypertrophy: Development of a novel in vitro multi-torsional stretch loading device
Source: PLoS One. 2022 Oct 21;17(10):e0275239. doi: 10.1371/journal.pone.0275239 (PMC9586365; doi:10.1371/journal.pone.0275239)
Supplement: Attachment — Submitted filename: Response to reviewers_v2.1.docx [file pone.0275239.s003.docx]

Answer to reviewers’ comments

Reviewer #1: (No Response)

Reviewer #2: This is an interesting work in introducing the new equipment and all my concerns are met. Looking forward to future application studies.

Thank you for taking your time to review this article and thank you for your positive comment, we appreciate it.

Reviewer #3:

1.As the experimental results, it can be seen that the device is effective, because it is a new method, we suggest that such a method of torsion and stretching should be compared with the more common FlexCell as possible.

Thank you for your comment. Our novel device provides three dimensional torsion/stress to the cell layers, which we believe is the key advantage in terms of mimicking the physiologic loading to LF cell in vivo. However, we agree to the reviewer’s idea that comparing the results from a widely accepted method can be useful in terms of reproducibility, therefore in future studies, once this device gains acceptance, the we will do compare the effectiveness of the device as recommended.

2.In vitro part, Fig. 6A is confused, why with the increase of time (0, 24, 48, 72h), there is no corresponding increase in the degree of confluence of LF cells.

Thank you for your comment. We believe the presented image shows significant difference of confluence by time difference, however as the reviewer feels differently, we have amended this figure by presenting an image from another duplicate experiment. We have changed the figure 6 as a whole, including 6A 72h, to be more representative and avoid any confusion.

3.The cytotoxicity/viability part should be proved by better and more commonly used methods, maybe due to some limitations, the method of lactate dehydrogenase release have been used to evaluate cytotoxicity, the cell biological effect of LDH should be explained.

Thank you for your comment. We agree with the reviewer’s comment as additional various cell viability testing would have resulted to a more fruitful result. However, this study was a study of a novel device, therefore we have started with a method that is rather familiar, reproducible and reliable, as suggested in previous peer-reviewed study **(We added the references as below**). We also agree with the reviewer that the manuscript needs the explanation on the background of cell biological effect of LDH. Therefore, we have added a section in materials and methods for the additional information as follows.

Added sentence: -

**Lactate Dehydrogenase Assay**

Lactate dehydrogenase (LDH), which is normally confined within the cell is released into culture supernatant after plasma membrane breakdown as result of cytotoxicity. This is a commonly used methods allowing simultaneous analysis of specimens rapidly and cost-effectively.(12) LDH was assayed using LDH assay kit (Roche, Mannheim, Germany) after various MSS loading – 0%, 5% and 15% - at various durations – 24, 48, and 72 hours.

Added reference: -

12.	Galluzzi L, Aaronson SA, Abrams J, Alnemri ES, Andrews DW, Baehrecke EH, et al. Guidelines for the use and interpretation of assays for monitoring cell death in higher eukaryotes. Cell Death & Differentiation. 2009;16(8):1093-107.

References advocated: -

Shin J, Hwang M, Back S, Nam H, Yoo C, Park J, et al. Electrical impulse effects on degenerative human annulus fibrosus model to reduce disc pain using micro-electrical impulse-on-a-chip. Scientific Reports. 2019;9(1):5827.

Hwang MH, Lee JW, Son H-G, Kim J, Choi H. Effects of photobiomodulation on annulus fibrosus cells derived from degenerative disc disease patients exposed to microvascular endothelial cells conditioned medium. Scientific Reports. 2020;10(1):9655

4.Mechanical stress is a key factor in LFH, In the in vitro part of the study of HLF, LF cells exhibits corresponding cellular phenotypic changes, such as increasing collagen I, collagen III and α-SMA, and there is no direct evidence for this in the author's paper, which does not seem to correspond to the HLF mentioned in the full text.

Thank you for your comment. We agree with the comment that for the direct evidence of the LFH, the full spectrum of cellular phenotypic changes, as suggested, as well as RNA expressions and intracellular signaling should be evaluated in a stepwise manner. However, in our previous study - Hur et al. Neurosurgery. 2015 and Kim et al. 2016 J Neurosurg Spine. 2016 – we discovered that VEGF and other angiogenesis, ECS regulation related signaling molecules and enzymes are related to LFH, acutally playing a role as the key initial step. As the former study is a comparison between LFH and non-LFH based on phenotypical characteristic, we proceeded with this study based on the target molecules from the former studies. We do believe that the current results represent the possibility of LFH in its current form, however, as the reviewer comments our future studies will include full spectrum of biologic assays describing the LFH.

The authors are aware of this limitation and therefore had included the following sentence in the discussion part; ‘We focused on the ligamentum flavum cellular protein production and expression and is currently lack of intracellular signaling RNA studies, and therefore cannot provide a comprehensive conclusion including subsequent cellular changes such as cytoskeletal remodeling or mechano-transduction signaling pathways. **Further studies concerning intracellular signaling assay, might lead us to a more information regarding the bio-mechanism of LFH.’**[Line 272-278]

Reviewer #4: The authors developed a multi-torsional mechanical stretch stress loading device and intended to evaluate its influence on the development of ligamentum flavum hypertrophy. The results showed that mechanical stress enhances the production of inflammatory cytokines and angiogenic factors.

Thank you for your comment

1. Line 186. Values unit ng/mL ± SE, which conflicts with the statement at line 140 in statistical analysis section. Need clarifications what were reported and please use consistent format throughout the manuscript.

Thank you for pointing out this in your comment. As pointed out, we have corrected the sentence from ‘Data are means ± standard deviations (SDs)’ to ‘Data are mean ± standard error (SE)’

Reviewer #5: In the manuscript, “Elucidating the effect of mechanical stretch stress on the mechanism of ligamentum flavum hypertrophy: Development of a novel in vitro multi-torsional stretch loading device”, by Kwon et al., the authors indicated that mechanical stress enhanced the production of inflammatory cytokines and angiogenic factors, and altered the expression of ECM-regulating enzymes, possibly triggering ligamentum flavum hypertrophy. Several major concerns (listed below) greatly reduce the significance of the work.

1. Why test LDH to react to cytotoxicity? CCK-8 should be used to reflect cellular activity.
Thank you for your comment. We agree with the reviewer’s comment as additional cell viability testing, such as CCK-8, would give more strength to the study result. However, this study was a study of a novel device, therefore we have started with a method that is rather familiar, reproducible and reliable, as suggested in previous peer-reviewed study.

The authors are aware of this limitation and therefore had included the following sentence in the discussion part; ‘We focused on the ligamentum flavum cellular protein production and expression and is currently lack of intracellular signaling RNA studies, and therefore cannot provide a comprehensive conclusion including subsequent cellular changes such as cytoskeletal remodeling or mechano-transduction signaling pathways. Further studies concerning intracellular signaling RNA with additional assay experiment, such as MTT assay, and various cell staining technique to validate cytoskeletal changes and cellular viability might lead us to a more abundant conclusion regarding the bio-mechanism.’ [Line 272-278]

2. LDH is released most at 15% stretch with 24h and 72h treatments. What explains the lower release at 15% stretch than at 5% stretch after 48 hours?
Thank you for your comment. Although, 5% stretch after 48 hours seem higher release than 15%, it is statistically insignificant. Given that the sample size is small it is unlikely that this reversed trend of LDH is meaningful. Another possible interpretation could be that the cell cytotoxicity is already present at a certain time point (after 48 hours) and further cell stress does not necessarily lead to a proportional increase of LDH release afterwards. However, we focused on statistically significant results, which correlated to the purpose of the experiment.

3. Figure 6B shows that 5% stretch treatment after 72 h is better than 48 h in terms of cell status, contradicting the authors' results.

Thank you for your comment. We believe the presented image shows significant difference of confluence by time difference, however as the reviewer feels differently, we have amended this figure by presenting an image from another duplicate experiment. We have changed the figure 6 as a whole, including 6A 72h, to be more representative and avoid any confusion.
